# Appropriate Allergic Rhinitis Medications Can Reduce Systemic Steroid Requirement and Prevent Rhinosinusitis

**DOI:** 10.3390/jcm13226809

**Published:** 2024-11-13

**Authors:** Min Kwang Byun, Won Jin Yang, Yong Jun Choi, Chi Young Kim, Jae Hwa Cho, Hoseob Kim, Jae-Hyun Lee, Jung-Won Park, Hye Jung Park

**Affiliations:** 1 Department of Internal Medicine, Gangnam Severance Hospital, Yonsei University College of Medicine, Seoul 06273, Republic of Korea; littmann@yuhs.ac (M.K.B.); octa821@yuhs.ac (W.J.Y.); cyj0717@yuhs.ac (Y.J.C.); cykim@yuhs.ac (C.Y.K.); jhcho66@yuhs.ac (J.H.C.); 2 Department of Data Science, HanmiPharm, Co., Ltd., Seoul 05545, Republic of Korea; hoseob.kim@hanmi.co.kr; 3 Division of Allergy and Immunology, Department of Internal Medicine, Yonsei University College of Medicine, Seoul 03722, Republic of Korea; jhleemd@yuhs.ac (J.-H.L.); parkjw@yuhs.ac (J.-W.P.); 4 Institute of Allergy, Yonsei University College of Medicine, Seoul 03722, Republic of Korea

**Keywords:** allergic rhinitis, medication, steroid, rhinosinusitis, COVID-19

## Abstract

**Background**: Allergic rhinitis (AR) is quite common and sometimes it requires systemic steroids and can be accompanied by coronavirus disease-2019 (COVID-19), rhinosinusitis, or asthma. We aimed to determine the comparative effect of different types of AR medications on clinical prognosis in real-world settings. **Methods**: We used national claims data provided by the National Health Insurance Service in the Republic of Korea. We enrolled 275,895 adult patients who were first diagnosed with AR and started AR medications between 1 January 2018 and 31 December 2018. We classified them into five groups according to the type of AR medication prescribed and analyzed their 3-year follow-up data. **Results**: The prescription rate of systemic steroids was low in the INCS group (19%), whereas it was 35–40% in other groups. INCS users needed less systemic steroids than other AR medication users (hazard ratio [HR], 0.503; 95% confidence interval [CI], 0.452–0.560; *p*-value < 0.001). The incidence of rhinosinusitis was approximately 11% in the other AR medication group and 6–8% in the other groups. AH (HR, 0.745; 95% CI, 0.616–0.903; *p* = 0.003), AH-LTRA (HR, 0.667; 95% CI, 0.551–0.808; *p* < 0.001), and INCS (HR, 0.746, 95% CI, 0.615–0.904; *p* = 0.003) significantly prevent rhinosinusitis, compared with other AR medication. However, other prognosis factors were not significantly correlated with the type of AR medications. **Conclusions**: INCS can reduce systemic steroid requirements and AH, AH-LTRA, and INCS prevent rhinosinusitis compared with other AR medications. As choosing an appropriate AR medication can determine the clinical outcomes, clinicians should be careful in prescribing proper AR medications.

## 1. Introduction

Allergic rhinitis (AR) is a relatively common disease, with a prevalence of 25% worldwide. Its symptoms include rhinorrhea, sneezing, nasal congestion, and pruritis. AR patients whose disease is not well controlled may experience tiredness, fatigue, sleep disturbance, and impaired quality of life [1]. Pharmacotherapy is frequently needed to control AR, and the recent worldwide guidelines [2,3]. recommend three drug categories to control AR symptoms: intranasal corticosteroids (INCSs), antihistamines (AHs), and leukotriene receptor antagonists (LTRAs). INCSs inhibit the influx of inflammatory cells and result in an improvement of inflammation, whereas AH helps reduce the hyperreactivity of the airways and increase the motility of epithelial cilia. LTRAs also reduce inflammation by inhibiting the leukotriene-related pathway’s effect on inflammatory cytokine cascades [4,5]. However, whether these drugs can also affect clinical outcomes remains unknown. In addition, there are few studies comparing the effects of AR medication on clinical prognosis [6].

AR frequently presents as a mild disease, however, it is not well controlled by classical AR medication sometimes, and it requires strong intervention. Although worldwide guidelines do not recommend systemic steroids [2], systemic steroid are prescribed in rare cases (2.6%) in real-world settings [7], and this may lead to various complications such as diabetes, cushingoid features, infections, anxiety, insomnia, hyperactivity, mood swings, reduced bone mineral density, and adrenal suppression, especially when it is used at a high dose and/or for a long time [8,9]. To avoid such complications, clinicians are trying to avoid and reduce the prescription of systemic steroids in AR [10]. Systemic steroid requirements in AR may also serve as a parameter to define its clinical outcomes, such as those used in asthma. If we can find specific AR medications that can reduce the systemic steroid requirement in this study, it will be helpful for clinicians choosing AR medication. This will finally lead to worldwide guidelines which discourage using systemic steroids.

AR is frequently accompanied by rhinosinusitis and asthma. Rhinitis, rhinosinusitis, and asthma are often considered as a continuum of the same underlying condition (typically type 2 inflammation) and can frequently coexist in the in the same patient [11,12]. This is because they may have a similar inflammatory process which appears in different parts of the united airway (so called “united airway disease”) [13,14]. There is an anatomical functional affinity between the nose, the paranasal sinuses, and bronchus [15]. AR represents an important risk factor for the development of chronic rhinosinusitis and asthma [16]. When AR is accompanied by asthma, the asthma is more severe compared with that without AR [17,18]. Nevertheless, there is little research confirming which AR medication can prevent the development of chronic rhinosinusitis or asthma.

In this explorative study, we aimed to determine the comparative effects of AR medications on clinical prognosis, including systemic steroid requirements, rhinosinusitis, asthma, and COVID-19 in AR patients by using real-world national data. We hypothesized that AR medications which are recommended in worldwide guidelines (AHs, LTRAs, and/or INCSs) will show superior effects to others (e.g., decongestants and/or intranasal antihistamines).

## 2. Materials and Methods

### 2.1. Ethics

This study was approved by the Institutional Review Board of Gangnam Severance Hospital (number: 3-2022-0095). The requirement for obtaining informed consent from the patients was waived owing to the minimal risk and retrospective nature of this study.

### 2.2. Data and Patients

In the Republic of Korea, the insurance system covers almost all Korean citizens, and all medical institutions claim their medical expenses [19]. We used this national claims data, provided by the National Health Insurance Service (NHIS), which includes information on all medical visits and the drug regimens prescribed by medical institutions [20], recorded between 1 January 2017 and 31 December 2020. Information that could identify individual participants was not accessed during or after data collection.

We defined AR patients as those who met both of the following criteria between 1 January 2018 and 31 December 2018: (i) ICD-10 codes for AR (J 30) and (ii) the administration of more than one of the following medications at least twice per year: AH, LTRA, INCS, intranasal AH, and decongestant (such as pseudoephedrine).

**Figure 1:**
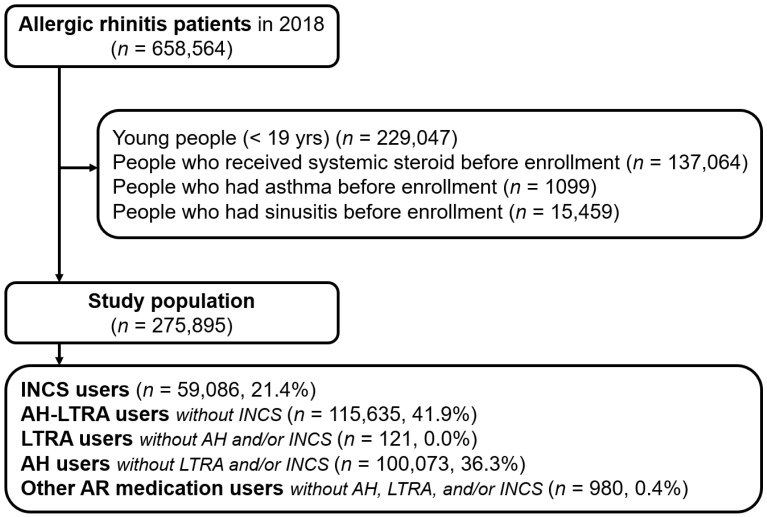
Study flow. INCS, intranasal corticosteroids; AH, anti-histamine; LTRA, leukotriene receptor antagonist; AR, allergic rhinitis.

Among the AR patients in 2018 (*n* = 658,564), we excluded individuals younger than 19 years old (*n* = 229,047), who received systemic steroids before enrollment (*n* = 137,064), and who had asthma (*n* = 1099) or rhinosinusitis (*n* = 15,459) before enrollment (“before enrollment” means from 1 January 2017 to 31 December 2017). We enrolled 175,895 AR patients and classified them into five groups: INCS users (*n* = 59,086; 21.4%), AH with LTRA (AH-LTRA) users without INCS (*n* = 115,635; 41.9%), LTRA users without AH and/or INCS (*n* = 121; 0.0%), AH users without LTRA and/or INCS (*n* = 100,073; 36.3%), and other AR medication users including intranasal AH and/or decongestant without AH, LTRA, and/or INCS (*n* = 980; 0.4%) (Figure 1).

### 2.3. Underlying Diseases

Hypertension, diabetes mellitus (DM), dyslipidemia, myocardial infarction (MI), stroke, and heart failure were defined using ICD-10 codes, and data within 1 year before enrollment (from 1 January 2017 to 31 December 2017) were used.

### 2.4. Charlson’s Comorbidity Index

Charlson’s comorbidity index (CCI), which predicts disease prognosis, was calculated to adjust for underlying diseases [19]. Well-known underlying conditions, which affect mortality and prognosis, were assessed using diagnostic codes from the claims data by using Quan’s coding algorithms [21].

### 2.5. Clinical Outcomes

The primary clinical outcome was AR prognosis, which included the prescription of systemic steroids and switching drugs to other AR medications. The referral of patients to other medical institutions was also assessed.

The secondary clinical outcomes were the development of rhinosinusitis, asthma, COVID-19, and all-cause mortality. Asthma was defined using ICD-10 codes and the history of asthma medication use, as used in a previous study [22]. Rhinosinusitis and COVID-19 were defined using their corresponding ICD-10 codes. All clinical outcomes were assessed from the diagnosis of AR until 31 December 2020, and we assessed the time point of the abovementioned clinical outcomes to use it in the time-varying Cox regression analysis.

### 2.6. Statistical Analysis

We performed ANOVA with a Bonferroni post-hoc test and chi-square test to compare continuous and categorical variables, respectively. Subsequently, we employed a Kaplan–Meier curve analysis and log-rank tests to determine the survival rate of clinical outcomes among groups. Finally, we generated a time variable from the first date of AR diagnosis to the date of the clinical outcomes. Considering this time variable, the hazard ratio (HR) and 95% confidence interval (CI) were calculated using Cox regression analysis to reveal the comparative effects of AR medications on clinical outcomes. Four models were evaluated: Model 1, unadjusted; Model 2, adjusted for age and sex; Model 3, adjusted for age, sex, income, and region; and Model 4, adjusted for age, sex, income, region, hypertension, DM, dyslipidemia, asthma, myocardial infarction, stroke, heart failure, and CCI. A time lag (14 days) was applied to define accurate clinical outcomes, and COVID-19 development was assessed from 1 January 2020. All data were analyzed using SAS Enterprise version 6.1 (SAS Institute Inc., Cary, NC, USA), and statistical significance was set at *p* < 0.05.

## 3. Results

### 3.1. Baseline Characteristics in the Study Population

Among the 275,895 AR patients enrolled in this study, AH-LTRA (41.9%) was the most frequently prescribed AR medication, followed by AH (36.3%) and INCS (21.4%). Other AR medications including intranasal AH and/or decongestant (*n* = 980; 0.4%) and LTRA (*n* = 121; 0.0%) were not frequently selected. The mean age of the enrolled patients was 36.9 years old. Male predominance (60.5%) was observed in the total population. The patients were more likely to live in cities (56.4% in total), and the mean CCI index was 0.12. Although there were statistically significant differences in all of the variables, there were no clinically meaningful differences between each group (Table 1).

**Figure 2:**
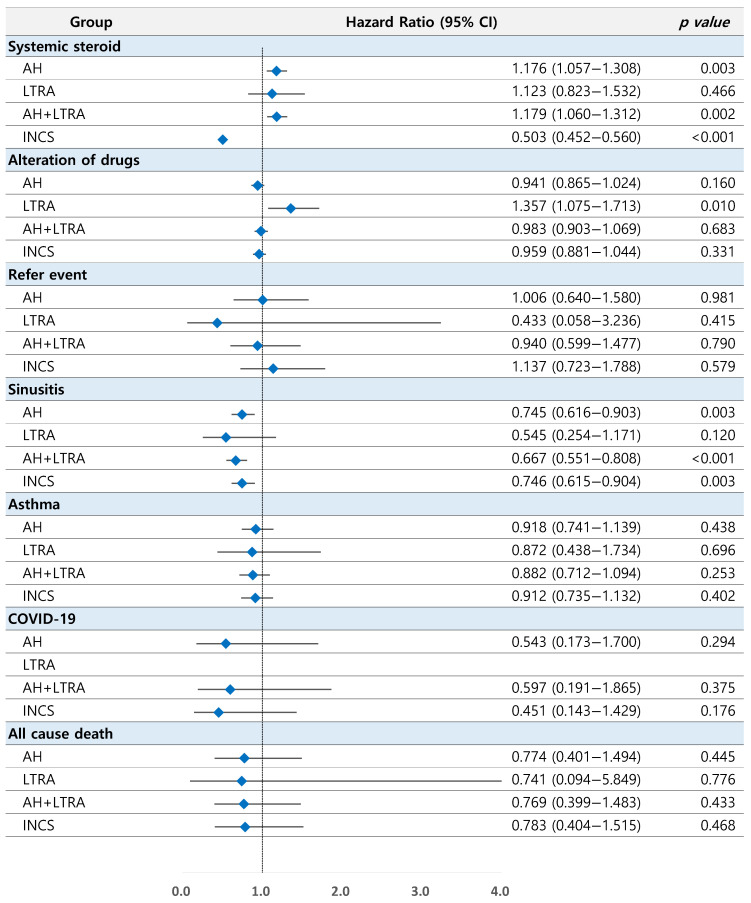
Forest plot for clinical outcomes compared to other AR medication group. AH, antihistamine; LTRA, leukotriene receptor antagonist; COVID-19, coronavirus disease; INCS, intranasal corticosteroid. Blue rectangles and the lines present hazard ratio and 95% CI.

**Figure 3:**
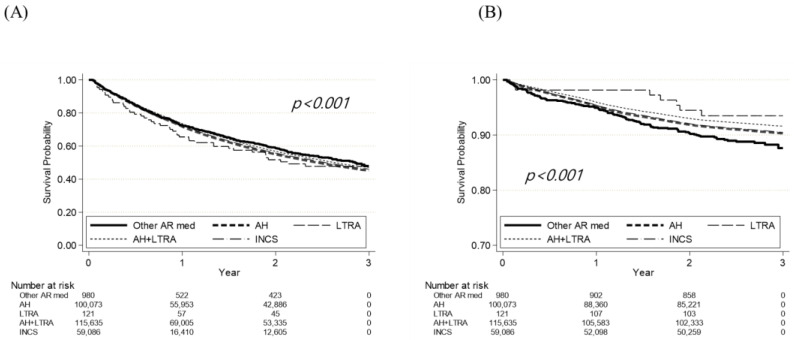
Kaplan–Meier graph for the systemic steroid prescription (**A**) and development of rhinosinusitis (**B**). AR, allergic rhinitis; AH, antihistamine; COVID-19, coronavirus disease; LTRA, leukotriene receptor antagonist; INCS, intranasal corticosteroid.

The prescription rate of systemic steroids was the highest in AH (40%) and AH-LTRA (40%) groups, followed by the LTRA (37%) and other AR medication (35%) groups and the INCS group (19%). INCS users needed less systemic steroid compared with other AR medication users (HR, 0.500; 95% CI, 0.449–0.557; *p* < 0.001 in the crude model and HR, 0.503; 95% CI, 0.452–0.560; *p* < 0.001 in the adjusted model). Compared with using other AR medications, using AH (HR, 1.177; 95% CI, 1.058–1.310; *p* = 0.003 in the crude model and HR, 1.176; 95% CI, 1.057–1.308; *p* = 0.003 in the adjusted model) and AH-LTRA (HR, 1.179; 95% CI, 1.060–1.312; *p* = 0.003 in the crude model and HR, 1.179; 95% CI, 1.060–1.312; *p* = 0.002 in the adjusted model) were significant high risk factors for systemic steroid requirements (Table 2, Figure 2 and Figure 3A).

Patients who used other AR medications at the first visit were switched to other drugs in 55% of the cases, and LTRA users were switched to other drugs most frequently (67%). The LTRA group showed a significantly higher risk of being switched to other drugs compared with the other AR medications group (HR, 1.330; 95% CI, 1.053–1.679; *p* = 0.017 in the crude model and HR, 1.357; 95% CI, 1.075–1.713; *p* = 0.010 in the adjusted model).

Regarding referral to other medical institutions, referral occurred in 2% of patients, regardless of the medication type. There was no significant difference in referral rates among drugs (Table 2 and Figure 2).

### 3.2. Other Clinical Outcomes According to the Medication Type

The incidence of rhinosinusitis was approximately 11% in the other AR medication group, and it was 6–8% in the other groups. The AH (HR, 0.767; 95% CI, 0.633–0.929; *p* = 0.007 in the crude model and HR, 0.745; 95% CI, 0.616–0.903; *p* = 0.003 in the adjusted model), AH-LTRA (HR, 0.677; 95% CI, 0.559–0.820; *p* < 0.001 in the crude model and HR, 0.667; 95% CI, 0.551–0.808; *p* < 0.001 in the adjusted model), and INCS (HR, 0.748; 95% CI, 0.617–0.907; *p* = 0.003 in the crude model and HR, 0.746, 95% CI, 0.615–0.904; *p* = 0.003 in the adjusted model) groups showed significantly lower risk of rhinosinusitis compared with other AR medications. Kaplan–Meier analysis showed significant differences in rhinosinusitis incidence according to the medication type (*p* < 0.001) (Table 3, Figure 2 and Figure 3B).

The incidence of asthma was 7–9%. COVID-19 occurred in 0.1–0.3% of AR patients. Regardless of the drug type, the all-cause mortality rate was 1%. The incidence of asthma, COVID-19, and all-cause mortality was not significantly related to the drug type for AR (Table 3 and Figure 2).

## 4. Discussion

To the best of our knowledge, this is the first large real-world explorative study, which revealed that choosing an appropriate AR medication can reduce systemic steroid requirements and prevent rhinosinusitis. We determined the comparative effects of AR medications on clinical prognosis in AR patients. We found that INCSs significantly reduced systemic steroid requirements, compared with other AR medications including intranasal AHs and/or decongestants. AH, AH-LTRA, and INCS prevented rhinosinusitis compared with other AR medications. This study illustrated that appropriate AR medication not only controls the symptoms of AR, but that it can also improve various clinical outcomes.

Our study revealed that INCS use significantly reduced the prescription rate of systemic steroids by approximately 50% compared with other AR medication use. Various AR medications are widely used to control AR in clinical practice; however, more than half of AR patients exhibited suboptimal rhinitis control in the real world, and some clinicians consequently prescribe systemic steroids to control AR (2.6%) [7]. This study showed higher prescription rate of systemic steroid (19–40%) than a previous study; however, we speculated that this may be due to the difference in study design: the previous study collected information on prescription rates at one specific point in time, whereas our study collected the history of prescriptions over several years. Systemic steroids can cause various complications [8]; therefore, the prescription rate of systemic steroids can be considered an important clinical outcome indicator. INCSs contain steroids as ingredients and are stronger than other drugs; therefore, they can reduce the prescription rate of systemic steroids. Then, INCSs are more strongly recommended compared with other medications in severe and persistent AR in worldwide guidelines [2]. We need to prefer INCSs to other drugs in AR patients at a high risk for systemic steroid use.

The incidence of rhinosinusitis was significantly reduced by 23–33% when AHs, AH-LTRAs, and INCSs were used compared with when other AR medications were used. Rhinitis and rhinosinusitis are often considered a continuum of the same underlying condition—only the site of the respiratory tract is different—and AR is a well-known risk factor for rhinosinusitis [16]. Unlike AR, which usually requires only simple symptom-controlling medications with few side effects, rhinosinusitis requires analgesics, INCSs, nasal irrigation, and sometimes even antibiotics or systemic steroids. In select cases, invasive procedures or surgery may be needed to control rhinosinusitis [23]. Therefore, AR medications that can prevent the development of rhinosinusitis suggest a clinical advantage for their use. We need to prescribe AHs, AH-LTRAs, and/or INCSs first to control AR in patients at a high risk for chronic rhinosinusitis rather than a simple decongestant and/or intranasal antihistamine.

Among the above AR medications, INCSs might affect nasal microbiota which represent the major environmental driver of the inflammatory process AR and rhinosinusitis. Dysfunctional interactions that occur between micro-organisms and the host immune system can trigger mucosal inflammation. It can lead to the alteration of the mucosal barrier, overgrowth of pathogens, susceptibility to infections, and finally, the development of rhinosinusitis [24]. Steroids (including INCSs) and antibiotics are well-known medications that alter the nasal microbiota. The protective effects of INCSs on the development of rhinosinusitis might be explained by these microbiota alteration mechanisms [25]. Among INCSs, newer agents, including mometasone furoate, fluticasone, and ciclesonide, are known to have a high affinity for the glucocorticoid receptor binding site and low systemic availability [26]. Although we could not classify the molecules in this study, a further study comparing the effects among different molecules will be helpful. In addition, the combination of INCS and intranasal antihistamine has shown great effects and is widely used in clinical practice [27]. Beyond a simple intranasal antihistamine, a combination with an INCS might be good choice.

The incidence of COVID-19 was not significantly different according to the type of AR medication. The nose has been considered the first entry point of viruses into the respiratory tract. Healthy nasal epithelial cells, as affected by AR medication, may be a protective factor against COVID-19. The entry of COVID-19 into epithelial cells requires the angiotensin-converting enzyme 2 (ACE2) and transmembrane serine protease 2 (TMPRSS2) [28]. The nasal expression of ACE2 and TMPRSS2 depends on age, allergic disease, and medication [29,30]. Some previous studies have shown positive effects of AR medication on COVID-19. Tuzer et al. revealed that INCS use prevents the loss of smell/taste during COVID-19 [31]. Another recent clinical study showed that INCS use can lead to better COVID-19 outcomes [32]. The in vitro study found that azelastine, which is an AH, can prevent and treat nasal colonization with the virus, and that it accelerated viral clearance in COVID-19 patients [33]. In a retrospective clinical study, azelastine use was significantly associated with lower COVID-19 severity [34]. LTRA use has also shown therapeutic potential to treat COVID-19 by interrupting errant signaling cascades [35,36]. Although we could not find significant differences in COVID-19 development in this study, a further long-term study might have significant findings.

We did not find significant differences in asthma incidence according to different AR drug types. AR is frequently associated with asthma (15–38%), and nasal symptoms are present in 6–85% patients with asthma [37]. In a 10-year retrospective study, a diagnosis of AR was significantly and independently predictive of developing asthma (odds ratio, 7.80) [38]. Asthma is more severe when accompanied with AR than without it [17,18]. However, the effects of AR medication in preventing asthma are not well-known. Although positive effects of immunotherapy in preventing the development of asthma in AR patients have been reported, there are limited data concerning general AR medications [39]. Further study with a larger number of patients and long-term follow-up data will be needed to define whether appropriate AR medication can prevent the development of asthma.

We found that AHs and AH-LTRAs significantly increased the prescription rate of systemic steroids by 19–20%. We speculate that this was because we could not collect information on AR severity, as it is highly likely that such medications were prescribed to people with relatively severe symptoms. Therefore, we did not consider these data to mean that the patients started taking steroids because they consumed AHs and AH-LTRAs but rather that the rate of steroid prescriptions increased because these patients originally had severe AR and were already on those medications. We also found that LTRA use increased the rate of switching to other drugs. This may also be because we did not adjust for the AR severity of AR. Since the LTRA group only included 129 patients, it is difficult to fully trust the LTRA data. Lastly, we found that a high proportion of AR patients (55–67%) wanted to change their medication over 3 years; however, only 2% of the AR patients changed their medical institutions. This means that many patients trusted their doctors and visited the same institute again, even if they deemed that the medication had little effect. Therefore, carefully selecting AR medication that suits the patient is of high importance.

This explorative study has several limitations as follows. First, owing to the use of national claims data, we only used diagnostic codes and medication history to define AR and other diseases. Second, we also could not assess the symptoms and severity of AR. Then, we could not adjust for them in the analysis. Third, the number of LTRA users without AH and/or INCS was relatively small compared with that of other medication users. Lastly, this study is retrospective, and it has fundamental limitations. Therefore, data concerning the LTRA group should be carefully interpreted.

## 5. Conclusions

In conclusion, we revealed that INCS use reduced systemic steroid requirements by approximately 50% compared with other AR medications, including only intranasal AHs and/or decongestants. AH, AH-LTRA, and INCS use significantly prevented rhinosinusitis by approximately 23–33% compared with other AR medications. Choosing an appropriate AR medication can determine the clinical outcomes; therefore, clinicians need to carefully prescribe proper AR medications. Further studies will be needed to find an association between AR medications and COVID-19 or asthma.

## Figures and Tables

**Table 1:** Baseline characteristics in study population.

		Total	Other AR Medication	AH	LTRA	AH-LTRA	INCS	*p*	*Cramer’s V or Cohen’s F*
Age (mean ± SD)		36.9 ± 16.7	38.0 ± 16.8	36.6 ± 16.7	40.2 ± 17.0	37.2 ± 16.9	37.0 ± 16.6	<0.001	<0.001
Sex	Male (%)	60.5	58.6	59.0	65.3	60.1	63.8	<0.001	0.037
Income	Low	19.5	18.4	19.1	12.4	19.7	19.7	<0.001	0.010
	Low-Med	17.8	17.4	17.6	17.4	17.8	18.2		
	Middle	20.7	19.9	20.5	25.6	20.6	21.1		
	High-med	18.2	17.7	18.2	14.9	18.4	18.0		
	High	22.1	25.3	22.8	27.3	21.8	21.2		
	Missing	1.8	1.3	1.9	2.5	1.7	1.8		
Region	City (%)	56.4	59.2	56.3	56.2	55.5	58.5	<0.001	0.0234
CCI (mean ± SD)		0.12 ± 0.41	0.12 ± 0.41	0.12 ± 0.41	0.12 ± 0.42	0.12 ± 0.40	0.12 ± 0.40	0.002	<0.001
Underlying disease (%)									
	Hypertension	6.7	5.8	6.7	7.4	6.8	6.6	<0.001	0.003
	DM	3.2	2.7	3.2	0.8	3.3	2.9	<0.001	0.010
	Dyslipidemia	2.1	1.7	2.1	3.3	2.1	2.1	0.777	
	MI	0.1	0.1	0.1	0.0	0.1	0.1	0.960	
	Stroke	0.6	0.7	0.6	0.0	0.6	0.6	0.846	
	Heart failure	0.1	0.1	0.1	0.0	0.1	0.1	0.177	
Number		275,895 (100%)	980 (0.4%)	100,073 (36.3%)	121 (0.0%)	115,635 (41.9%)	59,086 (21.4%)		

SD, standard deviation; AR, allergic rhinitis; INCS, intranasal corticosteroids; AH, antihistamine; LTRA, leukotriene receptor antagonist; DM, diabetes mellitus; MI, myocardial infarction.

**Table 2:** Cox regression analysis for clinical outcomes of allergic rhinitis.

Clinical Outcomes	Group	Event Number (Primary Endpoint 1)	Event Rate (%)	Duration (Year)	Incidence per 1000 Pys	Hazard Ratio (95% Confidence Interval) in Crude Model	*p*	Hazard Ratio (95% Confidence Interval) in Adjusted Model	*p*
Systemic steroid	Other AR medication	340	35	1970	172.6 (155.2–192.0)				
	**AH**	**39,606**	**40**	**193,652**	**204.5 (202.5–206.5)**	**1.177 (1.058–1.310)**	**0.003**	**1.176 (1.057–1.308)**	**0.003**
	LTRA	45	37	235	191.6 (143.1–256.6)	1.111 (0.814–1.517)	0.506	1.123 (0.823–1.532)	0.466
	**AH-LTRA**	**46,080**	**40**	**225,300**	**204.5 (202.7–206.4)**	**1.179 (1.060–1.312)**	**0.003**	**1.179 (1.060–1.312)**	**0.002**
	**INCS**	**11,384**	**19**	**133,795**	**85.1 (83.5–86.7)**	**0.500 (0.449–0.557)**	**<0.001**	**0.503 (0.452–0.560)**	**<0.001**
Alteration of drugs	Other AR medication	543	55	1519	357.4 (328.5–388.7)				
	AH	54,988	55	160,985	341.6 (338.7–344.4)	0.950 (0.873–1.034)	0.232	0.941 (0.865–1.024)	0.160
	**LTRA**	**81**	**67**	**168**	**482.6 (388.1–600.0)**	**1.330 (1.053–1.679)**	**0.017**	**1.357 (1.075–1.713)**	**0.010**
	AH-LTRA	66,199	57	186,396	355.2 (352.5–357.9)	0.986 (0.906–1.073)	0.739	0.983 (0.903–1.069)	0.683
	INCS	32,476	55	94,993	341.9 (338.2–345.6)	0.953 (0.876–1.038)	0.269	0.959 (0.881–1.044)	0.331
Referral	Other AR medication	19	2	2491	7.6 (4.9–12.0)				
	AH	1952	2	253,226	7.7 (7.4–8.1)	1.009 (0.642–1.585)	0.969	1.006 (0.640–1.580)	0.981
	LTRA	1	1	311	3.2 (0.5–22.8)	0.423 (0.057–3.160)	0.402	0.433 (0.058–3.236)	0.415
	AH-LTRA	2108	2	294,623	7.2 (6.9–7.5)	0.940 (0.598–1.477)	0.788	0.940 (0.599–1.477)	0.790
	INCS	1297	2	149,929	8.7 (8.2–9.1)	1.134 (0.721–1.784)	0.585	1.137 (0.723–1.788)	0.579

AR, allergic rhinitis; INCS, intranasal corticosteroid; AH, antihistamine; LTRA, leukotriene receptor antagonist. Adjusted for age, sex, income, region, and underlying disease. Significant values are presented as bold.

**Table 3:** Cox regression analysis for other clinical outcomes.

Clinical Outcomes	Group	Event Number (Primary Endpoint 1)	Event Rate (%)	Duration (Year)	Incidence per 1000 Pys	Hazard Ratio (95% Confidence Interval) in Crude Model	*p*	Hazard Ratio (95% Confidence Interval) in Adjusted Model	*p*
Rhinosinusitis	Other AR medication	106	11	2356	45.0 (37.2–54.4)				
	**AH**	**8402**	**8**	**243,110**	**34.6 (33.8–35.3)**	**0.767 (0.633–0.929)**	**0.007**	**0.745 (0.616–0.903)**	**0.003**
	LTRA	7	6	304	23 (11.0–48.2)	0.515 (0.240–1.107)	0.089	0.545 (0.254–1.171)	0.120
	**AH-LTRA**	**8636**	**8**	**284,438**	**30.4 (29.7–31.0)**	**0.677 (0.559–0.820)**	**<0.001**	**0.667 (0.551–0.808)**	**<0.001**
	**INCS**	**4856**	**8**	**144,375**	**33.6 (32.7–34.6)**	**0.748 (0.617–0.907)**	**0.003**	**0.746 (0.615–0.904)**	**0.003**
Asthma	Other AR medication	84	9	2379	35.3 (28.5–43.7)				
	AH	7898	8	243,944	32.4 (31.7–33.1)	0.958 (0.776–1.183)	0.6903	0.918 (0.741–1.139)	0.438
	LTRA	9	7	300	30.0 (15.6–57.7)	0.828 (0.418–1.639)	0.5874	0.872 (0.438–1.734)	0.696
	AH-LTRA	8790	8	283,956	31.0 (30.3–31.6)	0.918 (0.743–1.134)	0.427	0.882 (0.712–1.094)	0.253
	INCS	4594	8	144,796	31.7 (30.8–32.7)	0.941 (0.761–1.163)	0.5712	0.912 (0.735–1.132)	0.402
COVID-19	Other AR medication	3	0.3	979.2	1.5 (0.5–4.7)				
	AH	167	0.2	99,974	0.8 (0.7–1.0)	0.545 (0.174–1.707)	0.298	0.539 (0.172–1.687)	0.288
	LTRA	0	0	121	-	-	-	-	-
	AH-LTRA	218	0.2	115,512	0.9 (0.8–1.1)	0.616 (0.197–1.925)	0.404	0.609 (0.195–1.905)	0.394
	INCS	82	0.1	59,032	0.7 (0.6–0.9)	0.453 (0.143–1.434)	0.178	0.454 (0.144–1.439)	0.180
All causes of death	Other AR medication	9	1	2653	3.6 (1.9–6.9)				
	AH	710	1	270,524	2.8 (2.6–3.0)	0.774 (0.401–1.493)	0.444	0.774 (0.401–1.494)	0.445
	LTRA	1	1	332	3.2 (0.5–22.7)	0.889 (0.113–7.013)	0.911	0.741 (0.094–5.849)	0.776
	AH-LTRA	857	1	311,934	2.9 (2.7–3.1)	0.802 (0.414–1.539)	0.510	0.769 (0.399–1.483)	0.433
	INCS	422	1	159,847	2.8 (2.5–3.1)	0.775 (0.400–1.499)	0.449	0.783 (0.404–1.515)	0.468

AR, allergic rhinitis; INCS, intranasal corticosteroid; AH, antihistamine; LTRA, leukotriene receptor antagonist. Adjusted for age, sex, income, region, and underlying disease. Significant values are presented as bold.

## Data Availability

The ownership of the data used in this paper is held by the NHIS, not the authors. The data are publicly available and can be accessed and used directly for academic purposes at any time after approval from the NHIS. The website of the Health Insurance Data Sharing Service (https://nhiss.nhis.or.kr, accessed on 8 November 2024) is open to the public to access and use data.

## Abbreviations

AR	allergic rhinitis
INCS	intranasal corticosteroid
AH	antihistamine
LTRA	leukotriene receptor antagonist
DM	diabetes mellitus
MI	myocardial infarction
CCI	Charlson’s comorbidity index
CI	confidence interval
HR	hazard ratio
OR	odds ratio
COVID-19	coronavirus disease-2019
ICS	inhaled corticosteroid
ACE2	angiotensin-converting enzyme
TMPRSS2	transmembrane serine protease 2
NHIS	National Health Insurance Service
